# Changes of cardiac function: cardiac adaptation in patients with hypothyroidism assessed by cardiac magnetic resonance-a meta-analysis

**DOI:** 10.3389/fendo.2024.1334684

**Published:** 2024-06-11

**Authors:** Yucheng Yang, Chen Xue, Junyu Zhao, Laozhui Zhang, Yanwei Wang, Meixiang Ouyang, Ju Li, Haipeng Wang, Cuiyan Wang

**Affiliations:** ^1^Department of Radiology, Shandong Provincial Hospital Affiliated to Shandong First Medical University, Ji’nan, China; ^2^School of Medical Imaging, Binzhou Medical University, Binzhou, China; ^3^Department of Endocrinology and Metabology, The First Affiliated Hospital of Shandong First Medical University & Shandong Provincial Qianfoshan Hospital, Jinan, China; ^4^Department of Endocrinology, The Second People’s Hospital Of Dongying, Dongying, China; ^5^Department of Radiology, Shandong Provincial Hospital, Shandong University, Ji’nan, China

**Keywords:** hypothyroidism, levothyroxine, cardiac function, cardiac magnetic resonance, meta-analysis

## Abstract

**Objective:**

The meta-analysis aimed to explore the cardiac adaptation in hypothyroidism patients by cardiac magnetic resonance.

**Research methods and procedures:**

Databases including PubMed, Cochrane Library, Embase, CNKI, and Sinomed for clinical studies of hypothyroidism on cardiac function changes. Databases were searched from the earliest data to 15 June 2023. Two authors retrieved studies and evaluated their quality. Review Manager 5.4.1 and Stata18 were used to analyze the data. This study is registered with the International Platform of Registered Systematic Review and Meta-analysis Protocols (INPLASY), 202440114.

**Results:**

Six studies were selected for further analysis. Five of them reported differences in cardiac function measures between patients with hypothyroidism and healthy controls, and three studies reported cardiac function parameters after treatment in patients with hypothyroidism. The fixed-effect model combined WMD values for left ventricular ejection fraction (LVEF) had a pooled effect size of -1.98 (95% CI -3.50 to -0.44], *P*=0.01), implying that LVEF was lower in patients with hypothyroidism than in healthy people. Analysis of heterogeneity found moderate heterogeneity (*P* = 0.08, *I*² = 50%). WMD values for stroke volume (SV), cardiac index (CI), left ventricular end-diastolic volume index(LVEDVI), left ventricular end-systolic volume (LESVI), and left ventricular mass index(LVMI) were also analyzed, and pooled effect sizes showed the CI and LVEDVI of patients with hypothyroidism ware significantly decrease (WMD=-0.47, 95% CI [-0.93 to -0.00], *P*=0.05, WMD=-7.99, 95%CI [-14.01 to -1.96], *P*=0.009, respectively). Patients with hypothyroidism tended to recover cardiac function after treatment [LVEF (WMD = 6.37, 95%CI [2.05, 10.69], *P*=0.004), SV (WMD = 7.67, 95%CI [1.61, 13.74], *P*=0.01), CI (WMD = 0.40, 95%CI [0.01, 0.79], *P*=0.05)], and there was no difference from the healthy controls.

**Conclusion:**

Hypothyroidism could affect cardiac function, although this does not cause significant heart failure. It may be an adaptation of the heart to the hypothyroid state. There was a risk that this adaptation may turn into myocardial damage. Cardiac function could be restored after treatment in patients with hypothyroidism. Aggressive levothyroxine replacement therapy should be used to reverse cardiac function.

**Systematic review registration:**

https://inplasy.com, identifier (INPLASY202440114).

## 1 Introduction

Abnormal thyroid function usually refers to hyperthyroidism and hypothyroidism caused by various reasons, which are two common endocrine diseases in clinical practice. It has a serious impact on the functions of multiple organs and systems of the human body, usually manifested as abnormal levels of Thyroid hormones. These usually includes Triiodothyronine(T3), Thyroid hormone(T4), and thyroid-stimulating hormone (TSH). Meanwhile, it can be divided into overt and subclinical hypothyroidism by whether the levels of thyroid hormones are normal or not, and the latter only manifests as abnormal levels of TSH (1, 2). Current research shows that the cardiovascular system is one of the important target organs for thyroid dysfunction. Slight changes in thyroid function can have a major impact on the heart (3). Firstly, thyroid hormones exert an inotropic effect by regulating the expression of relevant genes in cardiomyocytes and regulating the activity of thyroid hormone receptor-α and thyroid hormone receptor-β (4–6). On the other hand, thyroid hormones promote the reuptake of calcium ions by the diastolic sarcoplasmic reticulum by regulating the levels of sarcoplasmic/endoplasmic reticulum calcium ATPase 2(SERCA2) and phosphorlamban(PLN) (7). That is essential for myocardial diastolic function. In addition, thyroid hormones also play a role in reducing peripheral resistance by promoting calcium reuptake in peripheral arterioles (8). So thyroid hormones also indirectly affect the heart by regulating cardiac preload and afterload (5).

Thyroid dysfunction was also thought to be associated with a number of cardiovascular diseases, including heart failure, atrial fibrillation, coronary atherosclerosis (8–14), and cardiovascular all-cause mortality (15). Therefore, a proper understanding of the impact of thyroid dysfunction on the myocardium can guide clinical practice and improve patient prognosis.

However, in the past, research in this area has usually relied on echocardiography, which is easy to operate. However the echocardiography is influenced by the subjectivity of different operators, and images are difficult to store, making it difficult to achieve accurate and objective follow-up. In addition, echocardiography can only obtain morphological data and cannot obtain myocardial histological features. CMR is a mature non-invasive examination method for obtaining cardiac morphological and histological features, which is less influenced by the operator. It can not only obtain large-field and high-resolution images to study myocardial morphology features but also obtain myocardial histological features (16). However, only a few studies have used CMR to explore the impact of thyroid dysfunction on the myocardium (17–23). Previous studies of thyroid dysfunction-related myocardial remodeling have focused on that in patients with hypothyroidism, and there have been fewer studies on myocardial remodeling due to hyperthyroidism (22, 23). In these studies, hyperthyroidism was thought to be associated with increased heart rate, myocardial hypercontractility, and left ventricular hypertrophy. Because eligible studies of myocardial remodeling in patients with hyperthyroidism were scarce (22, 23) and insufficient to complete a meta-analysis, this paper proposes a meta-analysis of cardiac changes associated with hypothyroidism based on the existing literature. Historically, studies based on echocardiography have usually assumed that hypothyroidism is associated with lower diastolic function. Results for changes in systolic function and myocardial hypertrophy in patients with hypothyroidism are inconsistent. As a more accurate tool than echocardiography, CMR is expected to clarify myocardial remodeling in patients with hypothyroidism.

## 2 Methods

### 2.1 Search strategy

This study is registered with the International Platform of Registered Systematic Review and Meta-analysis Protocols (INPLASY), 202440114. We searched five databases for clinical studies exploring the effects of hypothyroidism on the heart through CMR: PubMed, Cochrane Library, Embase, CNKI, and Sinomed for clinical studies of thyroxine in cardiac. Databases were searched from the earliest data to 15 June 2023 with the following search terms: (((((CMR) OR (cardiac magnetic resonance)) OR (cardiac MRI imaging)) OR (cardiac MR imaging)) OR (“Cardiac Imaging Techniques”[Mesh])) AND (((((((((((“Thyroid Gland”[Mesh]) OR (“Thyroid Diseases”[Mesh])) OR (“Thyroid Hormones”[Mesh])) OR (“Hyperthyroidism”[Mesh])) OR (“Graves Disease”[Mesh])) OR (“Hyperthyroxinemia”[Mesh])) OR (“Hypothyroidism”[Mesh])) OR (“Thyroid Dysgenesis”[Mesh])) OR (“Thyroiditis”[Mesh])) OR (“Thyroiditis, Autoimmune”[Mesh])) OR (“Hashimoto Disease”[Mesh])).

Inclusion criteria are that: (1) be written in English or Chinese. (2) report CMR data of patients with hypothyroidism, (3) clinical study. And exclusion criteria was poor methodological quality.

### 2.2 Study selection

Two authors screened studies independently. If their results were different, they would resolve their differences through discussion. If there was still controversy, a third author would be asked to reach a consensus.

### 2.3 Methodological quality assessment

The Newcastle–Ottawa Scale (NOS) was used to assess the methodological quality of case-control studies. The 9 points are considered the full score for the NOS, a score of 4 or less indicates “poor methodological quality”, and a scope of more than 4 is defined as “good methodological quality” (24). The others use the JBI critical appraisal tool to evaluate their quality. If more than 8 “YES” were obtained, the risk of bias was considered to below. Studies were considered to be at some risk of bias if they received 5 to 7 “YES”. Less than 5 “YES” were considered to be at high risk of bias (25, 26). Two authors assessed these items independently.

### 2.4 Statistical analysis

The outcome we needed was the difference in CMR parameters between the hypothyroidism and control groups. We also analyzed the change in CMR parameters before and after treatment. The weighted mean difference (WMD) and 95% confidence intervals were used for the pooled analysis. The fixed-effect model or random-effect model was used for the pooled analysis. *I*^2^ was used to evaluate heterogeneity. Study individuals were divided into overt hypothyroidism and subclinical hypothyroidism for subgroup analysis to reduce heterogeneity. Egger's test was used to evaluate publication bias. Review Manager 5.4.1 and Stata18 were used to perform the analysis.

## 3 Results

### 3.1 Search results and characteristics of included studies

**Figure 1:**
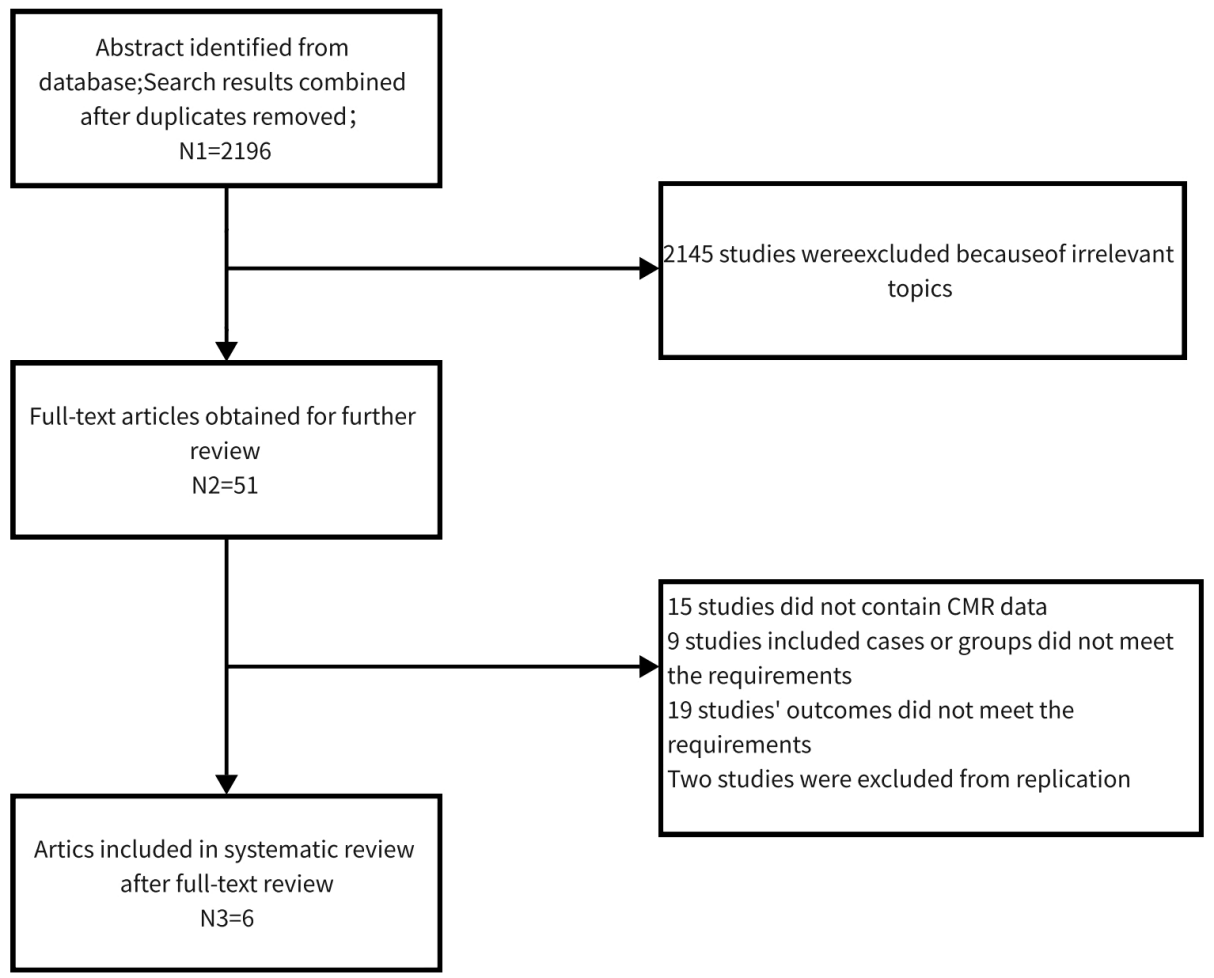
Flow chart of the systematic search process.

After retrieval from the above database, 51 articles of relevant studies were classified. After screening the full text, six articles were included in the systematic review. **Figure 1** shows the retrieval process. Three studies reported cardiac function parameters before and after treatment in patients with hypothyroidism (17, 20, 27). There were a total of 158 patients (both overt (19–21, 27) and subclinical (17–19) hypothyroidism) and 110 healthy individuals. The sample size ranged from 10 to 55 in the hypothyroidism group and 16 to 32 in the control group. Three studies (17, 20, 27) followed up patients with hypothyroidism after treatment. Four studies were from China one from Italy, and one from Germany. Except for Ripoli et al. (20) and BENGEL et al (27)., which used a 1.5T magnetic resonance system, all the other four studies used a 3.0T magnetic resonance. The characteristics of participants, gender radio, and age composition, the MR system are reported in **Table 1**.

**Table 1:** Characteristics of six included studies.

First author, year	Country	Characteristics of participants	Number of participants, n		Mean age, year		Follow-up time	MR system
			hypothyroidism	control	hypothyroidism	control		
Liu-2019 (19)	China	Overt and subclinical hypothyroidism	55	27	36.7 ± 8.3	35.1 ± 6.3	Not available	3.0T
Gao-2016 (18)	China	Overt hypothyroidism	23	30	36.60 ± 7.87	35.43 ± 8.25	174.75 ± 18.23	3.0T
Gao-2017 (17)	China	Overt hypothyroidism	24	17	36.7 ± 8.1	34.8 ± 9.1	90.64 ± 17.16	3.0T
Ripoli-2005 (20)	Italy	subclinical hypothyroidism	26	20	41.2 ± 6.9	38.4 ± 9.4	Not available	1.5T
Yao-2018 (21)	China	subclinical hypothyroidism	20	16	36.85 ± 8.46	35.56 ± 8.78	Not available	3.0T
BENGEL-2000 (27)	Germany	Overt hypothyroidism	10	0	50 ± 8	Not available	40 ± 13	1.5T

### 3.2 Quality assessment of included studies

The quality evaluation results of the case-control study and the single-arm study are shown in **Tables 2A**, **B**, respectively. Five studies were case–control studies (17–21). One study was a single-arm study (27). The quality of studies was assessed using NOS or JBI scales (28, 29). A study is considered to be of high quality if it scores five or more points on the NOS. All five studies were rated as five or higher. Although most studies only just achieved a score of 5, we still consider the quality of the included studies to be good. And the single-arm study was considered to be at some risk of bias.

**Table 2:** Quality assessment of five studies.

(A)																					
First author-year		Type of study design			Selection				Comparability				Exposure				Total				
Liu-2019 (19)		Case-control study			3				1				1				5			
Gao-2016 (18)		Case-control study			3				1				1				5			
Gao-2017 (17)		Case-control study			3				1				1				6			
Ripoli-2005 (20)		Case-control study			3				1				1				5			
Yao-2018 (21)		Case-control study			4				1				1				6			
(B)																					
First author-year		(1)		(2)		(3)		(4)		(5)		(6)		(7)		(8)		(9)		(10)	
BENGEL-2000 (27)		N		Y		Y		N		N		Y		Y		Y		N		Y	

### 3.3 LVEF

**Figure 2:**
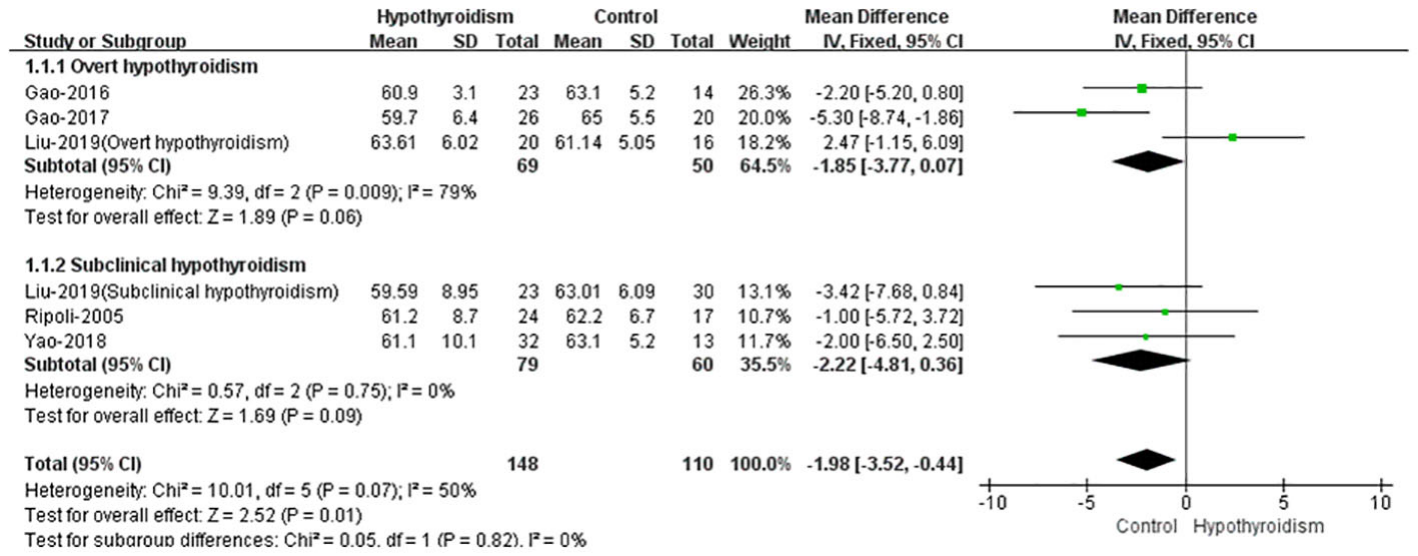
Forest plot of the LVEF through Hypothyroidism group and Control group.

Five studies reported the left ventricular ejection fraction (LVEF) of patients with hypothyroidism and control as an outcome (17–21). When comparing the LVEF of patients with hypothyroidism and healthy controls, four studies showed that there was no significant difference between the two groups (17–19, 21). Only one study found a significant difference in LVEF between the two groups (20), other studies found no significant difference in LVEF between the two groups. The fixed effect model was used to pooled WMD values of the LVEF, and the pooled results showed that the LVEF was significantly lower in hypothyroid group(WMD = -1.98, 95% CI [-3.50 to -0.44], *P* = 0.01). Analysis of heterogeneity showed moderate heterogeneity (Chi² = 10.00, *P* = 0.08, *I*² = 50%). When we classified the studies into overt and subclinical hypothyroidism, we found no statistically significant differences in LVEF in either overt or subclinical hypothyroidism (WMD = -1.85, 95% CI [-3.77 to -0.07], *P* = 0.06, WHD=-2.22 95% CI [-4.881 to 0.36], *P* = 0.09) (**Figure 2**). This may be due to the small number of included studies and some heterogeneity. However in most studies, there was a trend towards lower LVEF in patients in the hypothyroid group compared to the healthy control group, both in overt and subclinical hypothyroidism.

### 3.4 EDV and ESV

**Figure 3:**
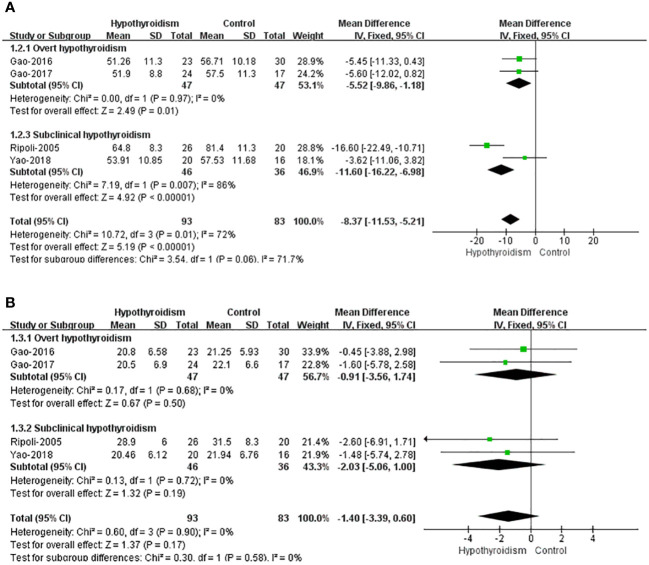
Forest plot of the LV Volume through Hypothyroidism group and Control group. **(A)** LVEDVI. **(B)** LVESVI.

Five studies reported left ventricular volume, four of which assessed left ventricular volume used left ventricular end-diastolic volume index (LVEDVI) and left ventricular end-systolic volume (LVESVI), and only Liu et al. used LVEDV and LVESV, so this study was not included in pooled analyses. All four studies reported a tendency for ventricular volume to decrease in patients with hypothyroidism compared with healthy controls, both LVEDVI and LVESVI, but only Ripoli et al. found a remarkable decrease in LVEDVI of patients with hypothyroidism. Fixed-effects model was used for pooled analysis, patients with hypothyroidism had significantly lower LVEDVI than healthy controls (WMD=-8.37, 95% CI [-11.53, -6.98], *P*=0.009), and heterogeneity analysis showed significant heterogeneity (Chi² = 10.72, *P*=0.01, *I*² = 72%). Subgroup analysis showed a notable difference in LVEDVI between individuals with subclinical hypothyroidism and healthy individuals (WMD = -11.60, 95% [-16.22, -6.98]), but heterogeneity was large (Chi² = 7.19, *P*=0.007, *I*² = 86%). Patients with overt hypothyroidism had significantly lower LVEDVI than healthy controls (WMD=-5.52 95CI% [-9.86, -1.18], *P*=0.01), and heterogeneity analysis showed no heterogeneity (Chi² = 0.00, *P*=0.97, *I*² = 0%). There was no clear difference in LVESVI between hypothyroidism with healthy controls (WMD=-1.40, 95% CI [-3.39 to 0.60], *P*=0.17). And there was no heterogeneity (Chi² = 0.60, *P*=0.90, *I*² = 0%) (**Figure 3**).

### 3.5 SV

**Figure 4:**
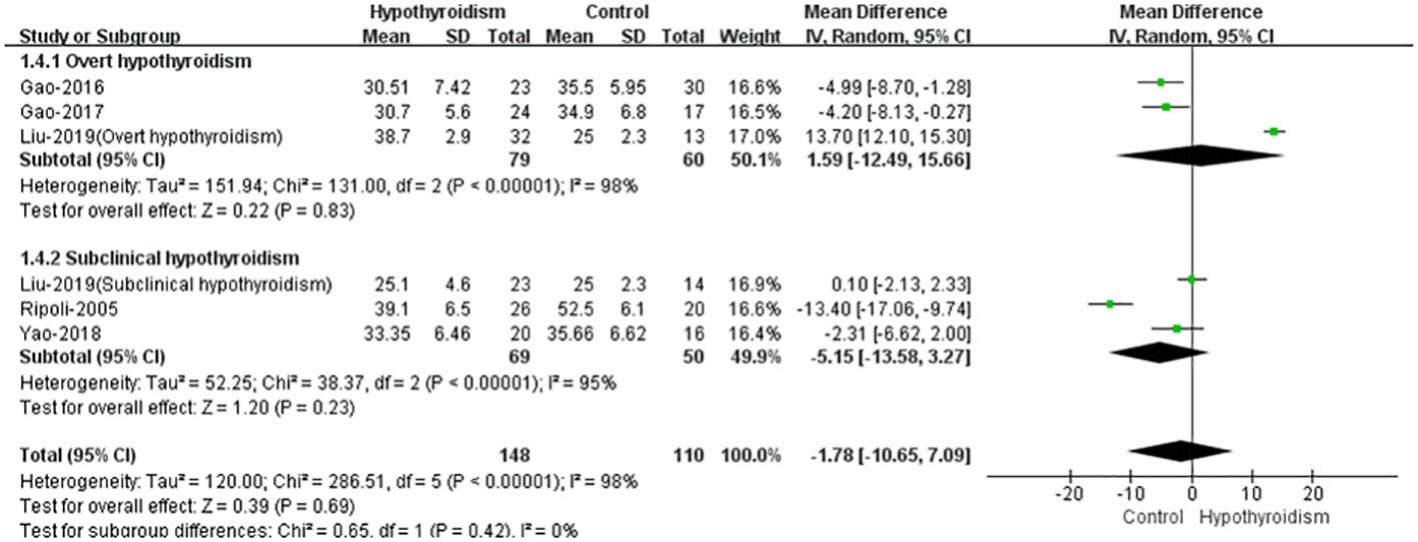
Forest plot of the SV through Hypothyroidism group and Control group.

Five studies reported stroke volume (SV) in both groups (17–21), except the study by Liu et al. (19), all studies found that SV and cardiac index (CI) were lower in patients with hypothyroidism, and three studies reported a statistical difference. The opposite result was obtained in the study by Liu et al., there was no significant difference in SV between two groups. Meanwhile they found that SV was higher in patients with overt hypothyroidism than in healthy controls. Using random-effects model for pooled analysis, there was no clear difference in SV between patients with hypothyroidism and healthy controls (WMD = -1.78, 95% CI [10.65 to 7.09], *P* = 0.69). The heterogeneity analysis showed large heterogeneity (Chi² = 286.51, P < 0.00001, I² = 98%). In subgroup analysis of subclinical hypothyroidism and overt hypothyroidism, there were no significant differences between healthy controls with subclinical or overt hypothyroidism in SV (WMD=-5.15, 95%CI [-13.58 to 3.27], *P*=0.23, WMD=1.59, 95%CI [-12.49 to 15.66], *P*=0.83, separately), both with large heterogeneity (Chi² = 38.37, *I*² = 95%, *P*<0.00001, Chi² = 131.00, *I*² = 98%, *P*<0.00001, separately) (**Figure 4**).

### 3.6 CI

**Figure 5:**
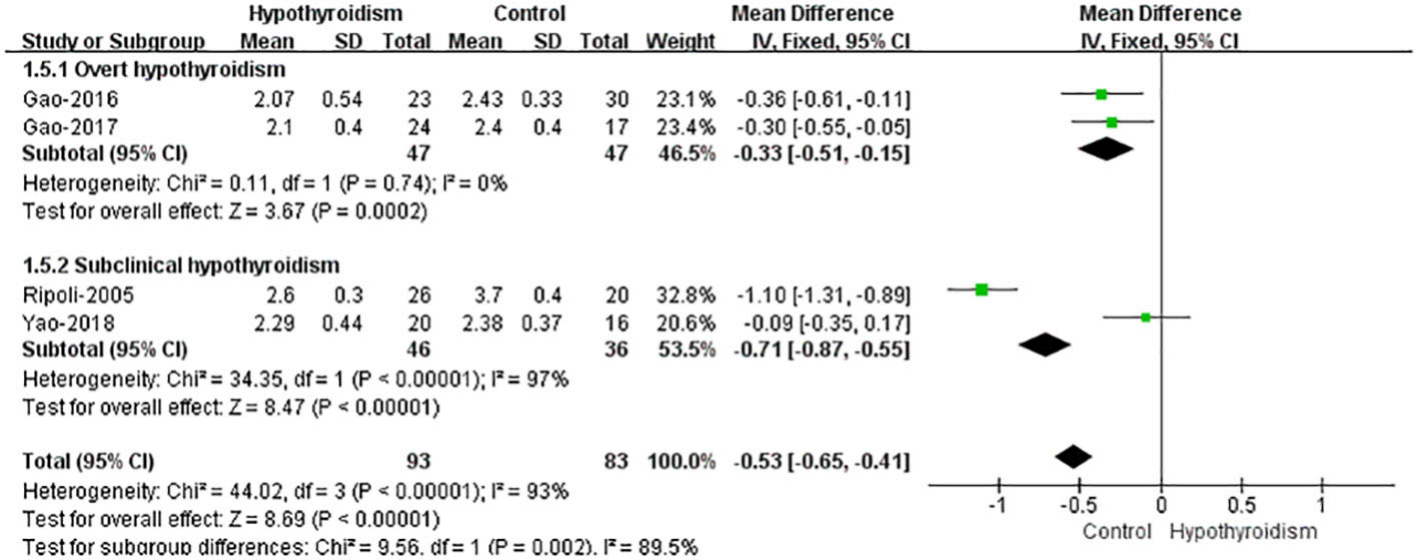
Forest plot of the CI through Hypothyroidism group and Control group.

Four studies (17, 18, 20, 21) reported CI data. Using fixed-effects model, the results showed that the patients with hypothyroidism had a marginally significant reduction in CI compared with healthy controls (WMD=-0.53, 95%CI[-0.65, -0.41], *P*<0.00001), and heterogeneity analysis showed large heterogeneity (Tau²=0.21, Chi²=44.02, *P* < 0.00001, *I*² = 93%). When patients were classified into subclinical hypothyroidism and overt hypothyroidism, both studies showed the lower CI in overt hypothyroidism group, which was consistent with the results of pooled analyses. And the fixed-effects model was used for pooled analysis, which showed significantly lower CIs in patients with overt hypothyroidism (WMD=-0.33, 95%CI[-0.51, -0.15], *P*<0.00001). And there was no heterogeneity (Chi²=0.11, *P*=0.74, *I*² = 0%). The study by Ripoli et al. showed that the CI of subclinical patients was significantly lower than that of the control group, while Yao et al. reported no significant difference in the CI between the two groups. Using fixed-effects model for pooled analysis, there was a remarkable decrease in CI of individuals with subclinical hypothyroidism compared with healthy controls (WMD = -0.71, 95% CI [-0.87 -0.55], *P*<0.00001), but heterogeneity analysis showed large heterogeneity in both studies (Chi²=34.35, *P*<0.00001, *I*²=97%) (**Figure 5**).

### 3.7 LVMI

**Figure 6:**
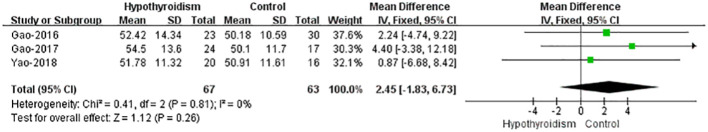
Forest plot of the LVMI through Hypothyroidism group and Control group.

Only three studies reported the data of left ventricular mass index (LVMI), and the results showed that no significant difference in LVMI between healthy controls and hypothyroidism group, either alone or pooled (WMD = -2.45, 95%CI [-6.73 to 1.82], P=0.26). The heterogeneity analysis showed no heterogeneity (Chi² = 0.41, P = 0.81; I² = 0%) (**Figure 6**). This means that the presence of hypothyroidism has no significant effect on LVMI.

### 3.8 Follow-up after levothyroxine treatment

Three studies followed up after levothyroxine treatment. Most studies reported recovery of cardiac function markers after levothyroxine replacement therapy. Using a random-effects model, LVEF (WMD = 6.37, 95%CI [2.05, 10.69], *P*=0.004), SV (WMD = 7.67, 95%CI [1.61, 13.74], *P*=0.01), CI (WMD = 0.40, 95%CI [0.01, 0.79], *P*=0.05) was significantly elevated in patients with hypothyroidism treated with Levothyroxine, but heterogeneity analysis showed high heterogeneity (Chi² = 6.09, *P* = 0.05, *I*² = 67%, Chi² = 8.03, *P* = 0.02, *I*² = 75%, Chi² = 6.06, *P* = 0.01, *I*² = 83%). Because the Bengel et al. study lacked a healthy control group, the post-treatment patients versus healthy control analysis were not included. After treatment, there were no differences in LVEF(WMD = 1.00, 95%CI [-1.76, 3.76], *P*=0.48), LVEDVI(WMD =-3.60, 95%CI [-8.28, 1.07], *P*=0.13), LVESVI(WMD = -2.28, 95%CI [-5.21, 0.66], *P*=0.89), SV (WMD = -2.39, 95%CI [-5.30, 0.53], *P*=0.11)in patients with hypothyroidism and healthy controls, and no significant heterogeneity was shown in heterogeneity analysis(Chi² = 0.18, *P* = 0.67, *I*² = 0%, Chi² = 0.01, *P* = 0.93, *I*² = 0%, Chi² = 0.02, *P* = 0.89, *I*² = 0%, Chi² = 0.82, *P* = 0.36, *I*² = 0%) (**Table 3**). Complete forest plot could be obtained in **Supplementary Figures 1–9**.

**Table 3:** Results of analyses of cardiac function after treatment in patients.

(A) After treatment VS Before treatment				
	Statistical methods	WMD【95%CI】	P	I²
LVEF	Random-effects model	6.37 [2.05, 10.69]	0.004	67%
SV	Random-effects model	7.67 [1.61, 13.74]	0.01	75%
CI	Random-effects model	0.40 [0.01, 0.79]	0.05	83%
LVEDVI	Random-effects model	7.39 [-1.86,16.66]	0.12	77%
LVESVI	Random-effects model	-0.86 [-3.75,2.03]	0.56	22%
(B) After treatment VS Controls				
	Statistical methods	WMD【95%CI】	P	I²
LVEF	Fixed-effects model	1.00 [-1.76, 3.76]	0.48	0%
SV	Fixed-effects model	-2.39 [-5.30, 0.53]	0.11	0%
LVEDVI	Fixed-effects model	-3.60 [-8.28, 1.07]	0.13	0%
LVESVI	Fixed-effects model	-2.28 [-5.21, 0.66]	0.89	0%

### 3.9 Publication bias

Egger's test was used to evaluate publication bias. Detailed results are shown in **Supplementary Tables 1–3**. Most subgroup analyses without significant publication bias. But the publication bias was found in the subclinical hypothyroidism subgroup regarding LVEDVI. Publication bias was also found in the pooled analyses on CI, but not in the subgroup analyses on overt hypothyroidism. In all analyses of cardiac function after hypothyroidism treatment, publication bias was found only in the analyses of LVEF and CI.

## 4 Discussion

According to our search results, this is the first meta-analysis of CMR studies of changes in cardiac function caused by hypothyroidism. This meta-analysis summarized the cardiac effects of hypothyroidism, including subclinical hypothyroidism and overt hypothyroidism. Four conclusions were drawn: 1) Cardiac function was reduced in patients with hypothyroidism compared with healthy individuals. 2) Cardiac function changes were more severe in patients with overt hypothyroidism than in those with subclinical hypothyroidism, but usually remained within normal limits. 3) Hypothyroidism did not have a significant effect on myocardial mass index, suggesting that the effect of hypothyroidism on cardiac function may be primarily functional rather than organic. 4) The effect of hypothyroidism on cardiac function was reversible. In summary, this hypothyroidism-induced functional reversible decrease in cardiac function within the normal range was more like a kind of cardiac adaptation phenomenon.

Many cardiovascular diseases were thought to be associated with the functional state of the thyroid gland. Thyroid dysfunction has been reported to impair myocardial contractile and diastolic function, especially diastolic function. This will increase the patient’s susceptibility to heart failure, thereby affecting the patient’s long-term prognosis. Subclinical hypothyroidism was also thought to be associated with coronary heart disease (8–14). However, most studies had small sample sizes, and some had mixed results. This article will focus on the effects of hypothyroidism on the heart.

CMR is a method that provides information on morphology, function, tissue, and even perfusion of the heart (16). Compared with echocardiography, cardiac magnetic resonance could provide not only the morphological and functional indicators of the heart, but also the tissue and perfusion characteristics of the heart. However few studies reported the tissue or perfusion characteristics of the heart in patients with hypothyroidism, this meta-analysis did not analyze relevant indicators and focused on morphology and function. Nevertheless, with the superior image quality of CMR, more accurate parameters of cardiac function could be obtained than those based on echocardiography. We believed that these data were still more informative than previous studies based on echocardiography.

This meta-analysis didn’t demonstrate significant difference in cardiac function in patients with subclinical hypothyroidism. Although a significant difference was found in the combined values of LVEDVI and CI in patients with subclinical hypothyroidism, they were unreliable because of their significant heterogeneity. Subclinical hypothyroidism was previously thought to be associated with cardiovascular diseases such as coronary heart disease (30–32). It does not conflict our findings. Cardiac disease previously thought to be associated with subclinical hypothyroidism often has a chronic course. The subclinical hypothyroidism patients in our included studies were usually diagnosed for the first time. Patients with subclinical hypothyroidism may have only mild cardiac changes because of the relatively short course of the disease. It is consistent with our results, as most studies showed a trend towards a reduction in LVEF (19, 20), CI (20, 21), and SV (20, 21) reduction in patients with hypothyroidism was found in most studies, although most studies did not show a significant difference. In addition, the pathological mechanism of myocardial injury induced by subclinical hypothyroidism has often been considered indirect, such as increased blood pressure and elevated lipids (33, 34). This also leads to a certain lag in changes in cardiac function.

This means that in addition to the direct effects of T3 and T4, TSH may also be involved in the process of cardiac function changes. Previous studies have found that patients with subclinical hypothyroidism with higher TSH had a higher risk of coronary heart disease and coronary heart disease death (35). However, initiating levothyroxine therapy in patients with subclinical hypothyroidism did not significantly reduce the risk of adverse cardiovascular events (36, 37). The cardiac effect of isolated elevated TSH and the need for aggressive intervention in patients with subclinical hypothyroidism require more prospective studies with large samples in the future.

This meta-analysis analysis found significant reductions in CI and LVEF in patients with hypothyroidism. Although LVEF did not decrease below the normal range in most patients, CI was slightly lower than normal in patients with hypothyroidism (38), Furthermore, CI was significantly lower in patients with overt hypothyroidism than in healthy controls. It suggested that the ejection reduction in patients with overt hypothyroidism. A recent study based on echocardiography reached the same conclusion (39). They believe that both systolic and diastolic function were reduced in hypothyroidism group. Similar results have been reported in animal experiments, with LVEF being significantly lower in mice of hypothyroid group than that of control group (40). In their study, dilation of the left ventricle and thinning of the left ventricular wall were observed simultaneously. However, these findings are inconsistent with the results of our analysis, and we did not observe any significant changes in the LVMI. Similarly, other studies based on echocardiography also found no difference in LVMI (39). Still other studies have found cardiac hypertrophy due to hypothyroidism (41). This may be due to the differences in blood pressure changes between hypothyroid patients and hypothyroid mice. It has been found that blood pressure in hypothyroid rats were significantly lower than those of controls at six weeks, and heart weight were also significantly reduced (42). However, blood pressure was usually elevated in patients with hypothyroidism (43, 44). Four studies (17, 18, 20, 21) in this meta-analysis reported data on blood pressure, and pooled values all found elevated blood pressure in people with hypothyroidism (systolic blood pressure: WMD=-3.78, 95% CI [-0.98 to -0.57], *P*=0.02, Chi² = 2.99, *P*=0.39, *I*² = 0%, diastolic blood pressure: WMD=-3.63, 95% CI [-5.74 to -1.52], *P*=0.0008, Chi² = 0.54, *P*=0.91, *I*² = 0%). It appears that the increase in peripheral resistance in humans with hypothyroidism may be more pronounced than the drop in cardiac output resulting in a net increase in systolic blood pressure. During this process, the relatively high myocardial mass was maintained in order to adapt to the increased peripheral resistance. This also may be attributed to the fact that the patients we included had less myocardial remodeling compared to mice in the hypothyroid group, and were still undergoing centripetal remodeling stage without significant changes in myocardial mass. Future detailed measurements of myocardial thickness in patients with hypothyroidism could help us uncover the underlying mechanism. This may indicate an adaptation of the heart to the hypothyroid state. Without timely intervention, patients with overt hypothyroidism may further develop into cardiac function impairment.

We did not find changes in LVESVI in either group. Our analysis revealed a reduction in LVEDVI in patients with overt hypothyroidism, which may suggest decreased cardiac diastolic function in patients with hypothyroidism The impaired diastolic function has been reported in hypothyroid patients (45, 46), and it is consistent with our analysis of LVEDVI. Decreased diastolic function has also been observed in recent animal experiments (47). Similarly, they also observed a slight enlargement of the left heart in some dogs of the hypothyroid group, but it also recovered after thyroid hormone supplementation. They attributed this phenomenon to a decrease in muscle strength in the hypothyroid dogs group. However, we only found this change in patients with overt hypothyroidism, and previous studies have suggested that ventricular diastolic function was impaired in patients with subclinical hypothyroidism (46).

The studies in this article didn’t include heart rate variability results, so heart rate changes in patients with hypothyroidism were not analyzed. Although there was no significant difference in heart rate between two groups, most studies found that the average heart rate of patients with hypothyroidism was lower than that of healthy individuals (47). A recent meta-analysis analysis of heart rate variability in hypothyroidism supports this view (48). This may result from the rebalancing of the vagus and sympathetic innervation (49). It can be speculated that decreased CI and LVEF in hypothyroid patients may be the result of a combination of low EDV and decreased heart rate and reduced ejection.

No differences in LVMI were found between patients with hypothyroidism and healthy individuals, which may indicate that changes in cardiac function were merely a cardiac adaptation. And the cardiac function restored in patients with hypothyroidism after thyroid hormone supplementation (41, 50, 51). Some studies found the reversal of ventricular hypertrophy after treatment (51). These findings support this view. However, some animal experiments have found elevated myocardial fibrosis markers in hypothyroid animals, and fibrosis of myocardium has been found in pathological examination (40). CMR Mapping technology and ECV are non-invasive means to detect myocardial fibrosis, At present, only three studies have reported the results of myocardial T1 mapping, all of which show that myocardial mapping values in patients with hypothyroidism are higher than those in healthy people (18, 19, 21), and myocardial fibrosis was a serious cause in cardiac function damage. This suggests that hypothyroidism may also lead to more severe myocardial damage. More research on CMR to detect myocardial injury in patients with hypothyroidism will help doctors and researchers reveal the role of myocardial fibrosis in this regard (52).

Patients with overt hypothyroidism have more severe cardiac function changes than subclinical patients. This suggests that measures should be taken to prevent the progression of subclinical hypothyroidism to protect cardiac function. Most studies have found that cardiac function indicators in patients with hypothyroidism recover after treatment with levothyroxine (17, 20, 27), and there is no significant difference with cardiac function indicators in healthy individuals (17, 20), indicating that cardiac adaptation caused by hypothyroidism is a reversible process. The results of studies on myocardial fibrosis suggest that this cardiac condition carries a risk of turning into myocardial injury (18, 19, 21, 40). Patients with hypothyroidism should be aggressively treated with levothyroxine replacement therapy to reverse the decline in cardiac function.

## 5 Limitation

The number of included studies was small, which may have affected the accuracy of the meta-analysis. At the same time, because there were insufficient studies reporting on myocardial tissue characteristics for a meta-analysis, it is impossible to deeply explore the internal mechanism of cardiac injury caused by hypothyroidism. Some studies were heterogeneous. Most of the studies had low NOS scores, although a score of 5 was basically consistent with the mate-analysis. Future high-quality studies of sample size will help to verify the above conclusions, and more reports on the characteristics of myocardial tissue will help to explore the underlying mechanisms. Publication bias was found in some analyses, but this was not found in the subgroup analyses of overt hypothyroidism. Therefore, we do not believe this bias has an impact on the main results.

## Data availability statement

The original contributions presented in the study are included in the article/**Supplementary Material**. Further inquiries can be directed to the corresponding authors.

## Author contributions

YY: Software, Methodology, Data curation, Writing – original draft. CX: Writing – original draft, Methodology, Data curation. LZ: Writing – original draft, Methodology, Data curation. YW: Writing – original draft, Methodology, Data curation. MO: Writing – original draft, Methodology, Data curation. JL: Writing – original draft, Methodology, Data curation. HW: Funding acquisition, Supervision, Project administration, Data curation, Writing – review & editing. CW: Writing – review & editing, Supervision, Project administration, Data curation. JZ: Writing – review & editing.
