# Supplementary material for: Changes of cardiac function: cardiac adaptation in patients with hypothyroidism assessed by cardiac magnetic resonance-a meta-analysis
Source: Front Endocrinol (Lausanne). 2024 Jun 11;15:1334684. doi: 10.3389/fendo.2024.1334684 (PMC11196803; doi:10.3389/fendo.2024.1334684)
Supplement: Supplementary file 1 [file DataSheet_1.docx]

Supplementary Material

**Supplementary Figure 1.** Forest plot of the LVEF through Euthyroid group and Hypothyroidism group.
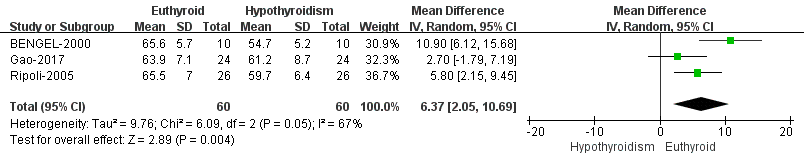
**Supplementary Figure 2.** Forest plot of the SV through Euthyroid group and Hypothyroidism group.
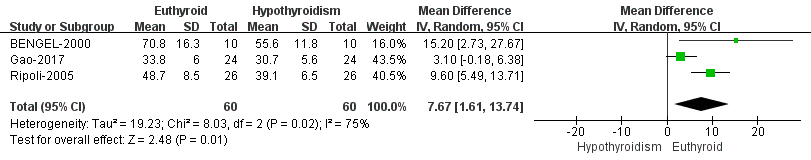
**Supplementary Figure 3.** Forest plot of the CI through Euthyroid group and Hypothyroidism group.
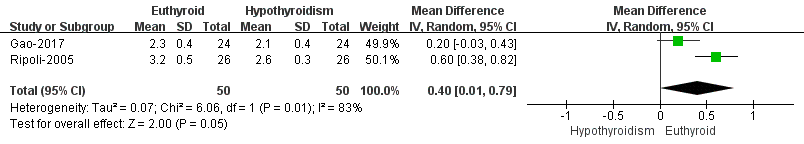
**Supplementary Figure 4.** Forest plot of the LVEDVI through Euthyroid group and Hypothyroidism group.
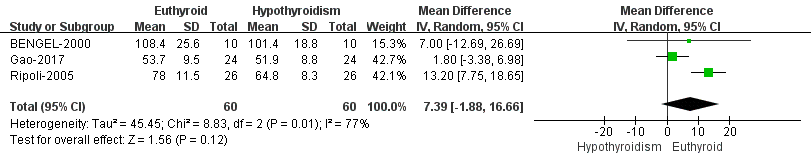
**Supplementary Figure 5.** Forest plot of the LVESVI through Euthyroid group and Hypothyroidism group.
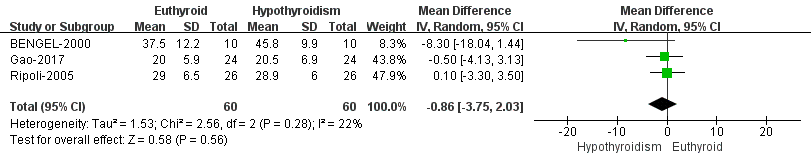


**Supplementary Figure 6.** Forest plot of the LVEF through Euthyroid group and Control group.
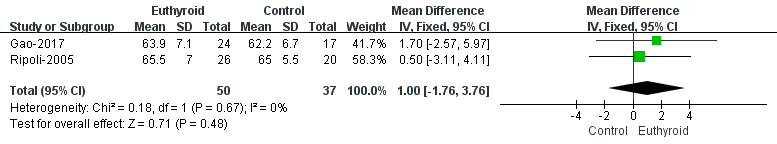


**Supplementary Figure 7.** Forest plot of the SV through Euthyroid group and Control group.
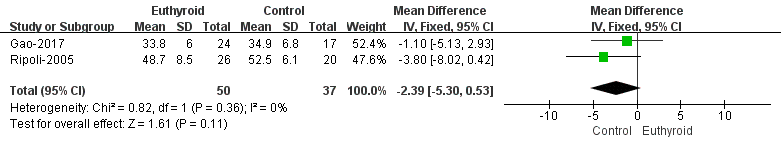


**Supplementary Figure 8.** Forest plot of the LVEDVI through Euthyroid group and Control group.
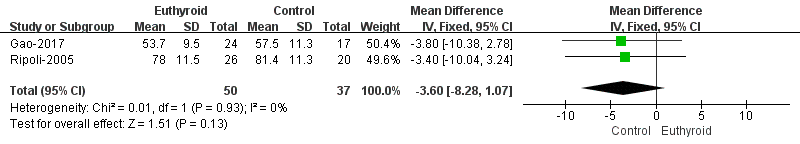


**Supplementary Figure 9.** Forest plot of the LVESVI through Euthyroid group and Control group.
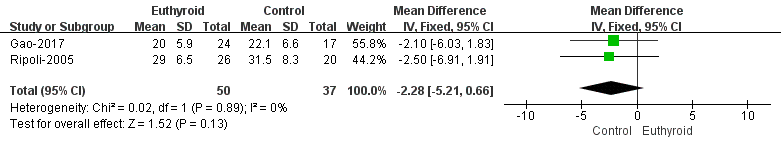


**Supplementary Table 1.** Egger’s test of “Hypothyroidism VS Control”.

|  | **Subgroups** | ***P* Value** |
| --- | --- | --- |
| LVEF | Overt hypothyroidism | 0.077 |
|  | Subclinical hypothyroidism | 0.4718 |
|  | ALL | 0.2434 |
| LVEDVI | Overt hypothyroidism | 0.9023 |
|  | Subclinical hypothyroidism | 0.004 |
|  | ALL | 0.1055 |
| CI | Overt hypothyroidism | 0.8463 |
|  | Subclinical hypothyroidism | 0 |
|  | ALL | 0 |

**Supplementary Table 2.** Egger’s test of “Euthyroid VS Hypothyroidism”.

|  | ***P* Value** |
| --- | --- |
| LVEF | 0.0359 |
| LVEDVI | 0.7518 |
| LVESVI | 0.1524 |
| SV | 0.7943 |
| CI | 0.0254 |

**Supplementary Table 3.** Egger’s test of “Euthyroid VS Control”.

|  | ***P* Value** |
| --- | --- |
| LVEF | 0.7023 |
| LVEDVI | 0.8709 |
| LVESVI | 0.9948 |
| SV | 0.4513 |
